# Carbohydrate Binding Modules: Diversity of Domain Architecture in Amylases and Cellulases From Filamentous Microorganisms

**DOI:** 10.3389/fbioe.2020.00871

**Published:** 2020-07-31

**Authors:** Andika Sidar, Erica D. Albuquerque, Gerben P. Voshol, Arthur F. J. Ram, Erik Vijgenboom, Peter J. Punt

**Affiliations:** ^1^Department of Microbial Biotechnology, Institute of Biology Leiden, Leiden, Netherlands; ^2^Department of Food Science and Agricultural Product Technology, Faculty of Agricultural Technology, Universitas Gadjah Mada, Yogyakarta, Indonesia; ^3^Sun Pharmaceutical Industries Europe BV., Hoofddorp, Netherlands; ^4^Dutch DNA Biotech B.V., Utrecht, Netherlands

**Keywords:** carbohydrate-binding module, cellulase, amylase, CAZymes diversity, domain architecture, *Aspergillus*, *Streptomyces*

## Abstract

Enzymatic degradation of abundant renewable polysaccharides such as cellulose and starch is a field that has the attention of both the industrial and scientific community. Most of the polysaccharide degrading enzymes are classified into several glycoside hydrolase families. They are often organized in a modular manner which includes a catalytic domain connected to one or more carbohydrate-binding modules. The carbohydrate-binding modules (CBM) have been shown to increase the proximity of the enzyme to its substrate, especially for insoluble substrates. Therefore, these modules are considered to enhance enzymatic hydrolysis. These properties have played an important role in many biotechnological applications with the aim to improve the efficiency of polysaccharide degradation. The domain organization of glycoside hydrolases (GHs) equipped with one or more CBM does vary within organisms. This review comprehensively highlights the presence of CBM as ancillary modules and explores the diversity of GHs carrying one or more of these modules that actively act either on cellulose or starch. Special emphasis is given to the cellulase and amylase distribution within the filamentous microorganisms from the genera of *Streptomyces* and *Aspergillus* that are well known to have a great capacity for secreting a wide range of these polysaccharide degrading enzyme. The potential of the CBM and other ancillary domains for the design of improved polysaccharide decomposing enzymes is discussed.

## Introduction

Plant biomass contains lignocellulose and starch which represent the most abundant carbohydrate biopolymers in nature. Lignocellulose is a complex polymer mix composed of lignin, an aromatic polymer and two carbohydrate polymers, cellulose and hemicellulose. The main component is cellulose consisting of β-1,4-linked D-glucose. In plant cell walls, cellulose chains interact with each other through hydrogen bonds to form microfibrils (Pérez et al., 2002; Somerville, 2006). Like cellulose, starch is polymeric carbohydrate consisting of solely glucose molecules linked via α-glycosidic bonds, instead of β-glycosidic bonds. Starch is composed of a mixture of two polymers, amylose and amylopectin. Amylose is a linear polysaccharide composed of α-1,4-linked D-glucose. Amylopectin is also a polysaccharide composed of primarily linear α-1,4-linked D-glucose as the backbone with branches that are α-1,6-linked to the backbone (Buleon et al., 1998).

The degradation of the renewable biopolymers cellulose or starch into sugar monomers by industry has a great economic value for its use as a feedstock for the production of various value-added products, such as fuels, chemicals and foods (Zhu et al., 2016). In general, degradation of the polysaccharides into sugar monomers requires the synergistic action of several classes of carbohydrate-active enzymes (CAZymes). Amongst the CAZymes, the glycoside hydrolases (GHs) are the major enzyme family involved in the degradation of polysaccharides such as starch and cellulose (Janeček et al., 2014; Berlemont and Martiny, 2016; Nguyen et al., 2018). However, both cellulose and starch contain a fraction that resists hydrolysis and is indicated as the recalcitrant fraction (Jeoh et al., 2006). Full decomposition of the recalcitrant fraction cannot be obtained by available enzyme cocktails and thus improvement of enzyme performance is necessary (Leggio et al., 2015). One of the options to consider in improving GH characteristics toward the decomposition of the recalcitrant fraction is the engineering of chimeric GH by the addition of one or more carbohydrate*-*binding modules (CBM).

In the CAZy classification system, the CBMs are a large group of protein domains that are frequently found attached to GH enzymes (Lombard et al., 2014). The CBMs exist as single or multiple (duplicated) domains attached to the C- and/or N-terminus of the catalytic domain. They consist of a relatively small number of amino acids, ranging from approximately 30–200 amino acids. CBMs do not have catalytic activity but function as substrate binding modules (Boraston et al., 2004; Lombard et al., 2014). It has been suggested that the binding characteristic of a CBM improves catalytic function of the CAZymes through targeting the enzyme to the substrate and increasing substrate-enzyme proximity as well as disrupting the crystallinity of the insoluble substrate fraction (Arantes and Saddler, 2010; Reyes-Ortiz et al., 2013; Bernardes et al., 2019; Chalak et al., 2019). As a consequence, the removal of the CBM from the enzyme results in a decreased enzymatic activity (Arai et al., 2003; Cockburn et al., 2017) and a reduced enzyme stability (Bissaro et al., 2017; Courtade et al., 2018; Teo et al., 2019).

Originally, many CBMs were classified as cellulose binding domains (CBDs) because of their affinity for cellulose. The first of these domains was found appended to the cellobiohydrolase CBHI and cellobiohydrolase CBHII in the fungus *Trichoderma reesei* (Van Tilbeurgh et al., 1986; Tomme et al., 1988). Thereafter, similar CBDs but with different amino acid sequences were also found attached to the cellulases from *Cellulomonas fimi* (Gilkes et al., 1988). As several modules showed affinity to substrates other than cellulose but still met the criteria as carbohydrate binding domain, the nomenclature CBM was introduced to cover the large variety of carbohydrate binding domains. For example, CBM1 was used for the first discovered fungal CBDs and thereafter also for modules with related amino acid sequences. The CBM2 family contains the CBDs showing high similarity with the bacterial CBDs as found in *C. fimi* (Gilkes et al., 1988) and other CBM families followed based on their particular ligand specificity, amino acid similarity and structural characteristics (see review Boraston et al., 2004). Like cellulose binding domains, starch binding domains (SBDs) were initially found in fungi as a C-terminal domain of *Aspergillus niger* glucoamylase (Hayashida et al., 1982; Svensson et al., 1982) and were collected in the CBM20 family (Lombard et al., 2014). As of April 2020, 86 CBM families are listed in the CAZy database (Lombard et al., 2014).

Although it is already very well known that CBM domains are often associated with GHs and are suggested to have a role in enhancing the enzymatic degradation, only limited information is available related to how widely the CBMs are distributed within the GH classes and how these domains are organized in the GHs produced by a given microorganism. Multiple CBMs from different families can also be present in GHs at either the C- or N-terminus. It has been reported that the distribution of the polysaccharide degrading enzymes among the CAZy classes is highly variable within genera, including the GH families (Benoit et al., 2015; Berlemont, 2017). In that regard, mapping the CBM distribution within the GHs may provide novel insight for designing new enzyme architecture with the potential to improve polysaccharide degradation. One of the approaches is to design a chimeric enzyme containing one or more CBMs (Punt et al., 2011; Duan et al., 2017).

This review provides a comprehensive overview on the variation and distribution of CBM domains present in the GH families of cellulases and amylases from filamentous microorganism, particularly *Aspergillus* and *Streptomyces.* Filamentous microorganisms, such as *Streptomyces* (Book et al., 2016; Montella et al., 2017) and *Aspergillus* (Punt et al., 2002; Yuan et al., 2008; Benoit et al., 2015; Gruben et al., 2017) have a great potential to produce large numbers of CAZymes. Both genera are also well-known for the production of industrially important extracellular enzymes (Fleißner and Dersch, 2010; Sevillano et al., 2016). Many Aspergilli species, mainly the black *Aspergillus* strains such as *Aspergillus niger* and *Aspergillus oryzae* are used for the production of a variety of industrial enzymes such as amylases, pectinases, proteases, β-galactosidase, glucoamylase, lipase, phytase, protease, hemicellulase, and cellulase (Fiedler et al., 2013; Polizeli et al., 2016). *Streptomyces* strains are also used as production hosts for enzymes. *Streptomyces lividans* and *Streptomyces coelicolor* as the examples, have been applied for recombinant extracellular protein production, including hydrolases, proteases/peptidases, chitinases/chitosanases, cellulases/endoglucanases, amylases, and pectate lyases (Anné et al., 2014; Hamed et al., 2018; Ahmed et al., 2020). The striking variety of domain architectures in these organisms calls for a new approach toward enzyme engineering. Examples are discussed where the attention is focused on designing chimeric enzymes with *Streptomyces* and *Aspergillus* inspired domain organization for improved cellulose or starch degradation.

## Enzyme Families Involved in Starch and Cellulose Degradation

### Starch-Degrading Enzymes (α-Amylase, Gluco-Amylase, β-Amylase)

**FIGURE 1:**
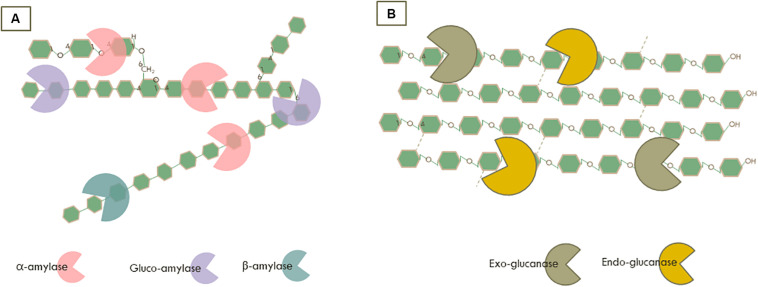
Synergistic action of the enzymatic degradation by **(A)** Amylases on starch and **(B)** Cellulases on cellulose.

All enzymes capable of degrading starch are collectively indicated as amylolytic enzymes. In this review, we focused on the main endo- and exo-amylolytic enzymes which are involved in saccharification of starch into glucose (Figure 1A). Endo-amylases, mainly α-amylases (1,4-α-D-glucan glucanohydrolase, EC 3.2.1.1) hydrolyse randomly the internal α-1,4-glucan chains to yield small oligosaccharides units (Gupta et al., 2003). Subsequently, gluco-amylases (1,4-α-D-glucan glucohydrolase, EC 3.2.1.3) as the main exo-amylolytic enzymes can hydrolyse linear α-(1,4) as well as the branching sites with an α-(1,6)-linkage at the non-reducing end of starch to release glucose (Sauer et al., 2000). Furthermore, β-amylases are another important class of exo-acting enzymes producing mainly maltose by cleaving starch molecules at the non-reducing end of α-1,4-glucan chains (Ray and Nanda, 1996). Their industrial application is essentially for maltose-rich syrup production (Niu et al., 2018). Overall, amylolytic enzymes have been largely used in a broad range of industrial applications, such as foods, detergents, pharmaceuticals, and the paper as well as textile industries (Ray and Nanda, 1996; de Souza and de Oliveira Magalhães, 2010).

In general, α-amylases are composed of three domains. Domain A has a TIM-barrel fold containing the active site residues and a chloride ion-binding site. Domain B is a long loop region that contains a calcium-binding site, and domain C is the C-terminal β-sheet domain that shows variability in sequence and length amongst amylases. Several amylases have at least one conserved calcium-binding site, as calcium is essential for enzyme stability, while the chloride binding site present in the active site region is important for the catalytic function. The active center of α-amylases, in general, involves mainly asparagine (Asp) and glutamic acid (Glu) residues (Pujadas and Palau, 2001; Mehta and Satyanarayana, 2016). For glucoamylase, the catalytic domain folds as a twisted (α/α)6-barrel, with the active site in a pocket shape at the N-terminal side of barrels. Two conserved glutamic acid residues are involved in the catalytic mechanism (Sauer et al., 2001; Aleshin et al., 2003). The β-amylase structure exhibits a well conserved (β/α) 8-barrel fold in the core domain with the active site as a deep cleft within the barrel where two glutamic acid (Glu) residues are involved in the hydrolysis (Mikami et al., 1994; Oyama et al., 2003).

In the sequence-based classification of GHs in the CAZy database, α-amylases are represented by four different families, GH13, GH57, GH119, GH126. The main α-amylase family, GH13, is found in a broad range of organisms, including bacteria, archaea, eukaryota, and even viruses. GH57 is found in bacteria, archaea, and very limited in eukaryotes. The other two families, GH119 and GH126 have been found in bacteria only (see review Janeček et al., 2014; Lombard et al., 2014). The glucoamylase family, GH15, does widely occur in prokaryotic and eukaryotic microorganisms including fungi (Aleshin et al., 2003), while the GH97 glucoamylases are predominantly found in bacteria (Lombard et al., 2014). Furthermore, the β-amylase family GH14 is in majority present in plants and bacteria (Lombard et al., 2014).

### Cellulose Degrading Enzymes (Exo-Glucanase, Endo-Glucanase)

Almost 50% of lignocellulose is composed of cellulose (Somerville, 2006; Isikgor and Becer, 2015). The degradation of cellulose is accomplished by multiple GHs which are typically acting together as a cocktail with complementary and synergistic modes of action (Payne et al., 2015). For example, endoglucanase (EC 3.2.1.4) cleaves randomly the internal β-glycosidic bonds of cellulose, while exo-glucanases/cellobiohydrolases cleave at both the non-reducing (EC 3.2.1.91) and reducing (EC 3.2.1.176) end of the cellulose chain to release soluble cellobiose (Figure 1B). These enzymes are used on a large scale for different industrial applications and are mainly produced by microorganisms, especially from the fungal and bacterial kingdoms.

Based on the active site topology, the catalytic machinery of cellobiohydrolases is set up in a tunnel-like conformation, enabling the single cellulose chain to be cleaved at the reducing or non-reducing terminus (Parkkinen et al., 2008). In contrast, the endoglucanase catalytic topology is shaped as an open cleft, allowing a linear amorphous cellulose chain to be degraded randomly at any part of the chain (Davies and Henrissat, 1995). Despite the fact that some enzymes share structural similarity, they may exhibit variations in the active site topology as well as the surface loops resulting in different substrate specificities. Based on structural modeling and enzymatic assays of GH6s from *Podospora anserina*, the exoglucanase PaCel6A has an active-site tunnel topology with the loop corresponding to amino acids 415–429 leading to the formation of the tunnel shape. In contrast the endoglucanase PaCel6B lacks the short 15 amino acid loop at the C-terminus and is predicted to contain a binding cleft rather than a tunnel structure. Those differences in the surface loop influence the mechanism of action toward substrates. Therefore, GH6 PaCel6B showed higher activity on carboxymethylcellulose (CMC) which is a highly specific substrate for endo-acting cellulase. CMC is decrystallized cellulose and thus contains more amorphous sites that are ideal for access to the cellulose chain by endoglucanases that cleave internally. Whereas, the exoglucanase GH6 PaCel6A showed higher activity on the insoluble microcrystalline cellulose Avicel than on CMC, as measured by the liberation of cellobiose from the non-reducing end of the cellulose chain (Poidevin et al., 2013). In addition, deletion of an exo loop in *C. fimi* cellobiohydrolase allows this enzyme to hydrolyze internal β-1,4-glucosidic bonds, altering its exolytic activity into endolytic activity (Meinke et al., 1995). Moreover, variations in the surface loop conformation were shown to determine the substrate specificity in GH5, such as exo- and endo-mannanase activity (Dias et al., 2004; Kumagai et al., 2015) as well as endoglucanase activity (Tseng et al., 2011). Therefore, in the CAZy classification enzymes could be clustered together as one GH family, although they are different in substrate specificity For example in members of the GH5 family a wide range of cellulase activities have been demonstrated: endo-β-1,4-glucanase, exo-β-1,4-glucanase, as well as non-cellulase activities: endo-β-1,4-xylanase, β-mannosidase, β-glucosylceramidase, glucan β-1,3-glucosidase, glucan endo-1,6-β-glucosidase, mannan endo-β-1,4-mannosidase, cellulose β-1,4-cellobiosidase, chitosanase, xyloglucan-specific endo-β-1,4-glucanase, endo-β-1,6-galactanase, β-1,3-mannanase, endo-β-1,3-glucanase/laminarinase, chitosanase, α-L-arabinofuranosidase (Lombard et al., 2014). In the CAZy database the endoglucanases are classified in 15 different families, GH5, GH6, GH7, GH8, GH9, GH10, GH12, GH26, GH44, GH45, GH48, GH51, GH74, GH124, and GH148. The exoglucanases are divided over 5 GH families, GH5, GH6, GH7, GH9, and GH48. Fungal cellulose degrading enzymes in these families commonly consist of a simple domain arrangement of only a catalytic domain or a catalytic domain with one accessory domain (Berlemont, 2017). Unlike the fungal cellulases, the bacterial cellulases often have a more complex domain structure which can also be involved in assembling multi-enzyme complexes, called cellulosomes (Bayer et al., 2004).

## Multi Domain Architecture and Distribution of CBMS in Starch and Cellulose Specific Glycoside Hydrolases From the Genus *Streptomyces* and *Aspergillus*

**FIGURE 2:**
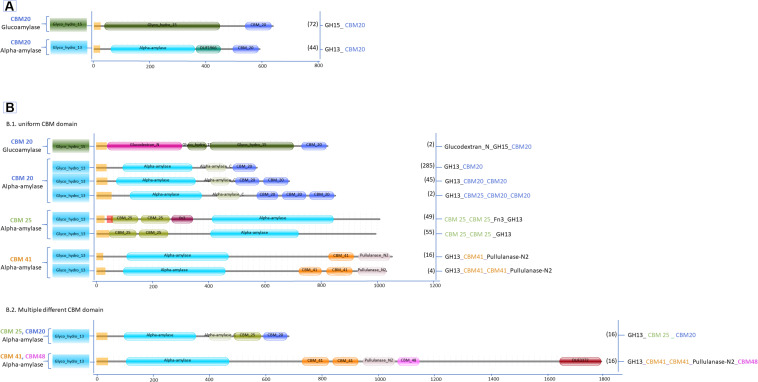
Domain organization of amylases carrying CBM **(A)** *Aspergillus*; **(B.1)** *Streptomyces* single CBM and **(B.2)** *Streptomyces* multiple CBM domains. Between brackets is the number of protein sequences in the various *Aspergillus* and *Streptomyces* species retrieved from the UniProtKB and the CAZy databases.

**FIGURE 3:**
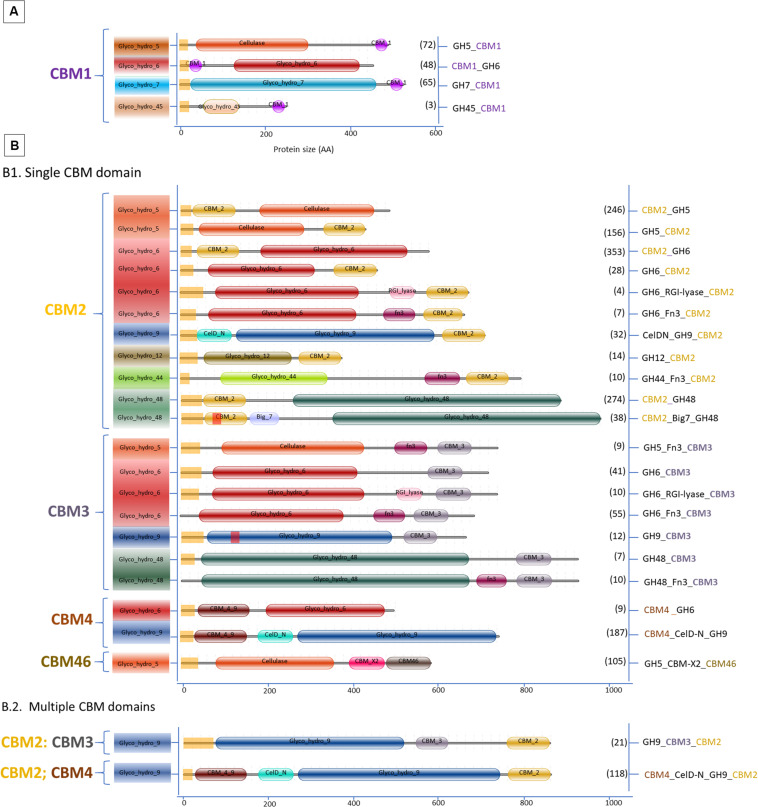
Domain organization of cellulases carrying CBM. **(A)** *Aspergillus*; **(B.1)** *Streptomyces* single CBM and **(B.2)** *Streptomyces* multiple CBM domains. Between brackets is the number of protein sequences in the various *Aspergillus* and *Streptomyces* species retrieved from the UniProtKB and the CAZy databases.

Many proteins within the various GH families show a distinct variation in the domain architecture. As discussed above, in particular the CBM domains are frequently associated with the GH catalytic domain. The abundance of GHs for cellulose degradation varies across bacterial phyla, of which the actinobacteria showed the most extensive variation in GH-cellulase content and are representatives of potential cellulose degraders (Berlemont and Martiny, 2015). For mapping the CBM and the GH diversity, datasets of cellulases and amylases carrying CBMs from *Aspergillus* and *Streptomyces* were retrieved from the UniProtKB and the CAZy databases. Subsequently, these datasets were grouped based on the type of CBMs and curated to avoid the inclusion of duplicated sequences by running protein alignment for each GH family in the same species by the Clustal Omega alignment program. Identical sequences from the same species were removed. Eventually, the final datasets were grouped based on the type of CBM associated with selected GHs. In addition, for constructing the graphical domain representation as presented in Figures 2, 3, signal peptides and transmembrane segments were predicted using Phobius (Käll et al., 2004). Subsequently, Pfam domains were determined using the HMMER program (version 3.2.1) against the Pfam database version 31.0 (Eddy, 2011; El-Gebali et al., 2019). Finally, the predictions were programmatically extracted and converted into detailed graphical representations of the various domain organizations, as was previously done for the Auxiliary Activities CAZymes, the Lytic Polysaccharide Mono Oxygenases (LPMO) (Voshol et al., 2017, 2019).

In general, filamentous fungi represent a richer reservoir of CAZymes compared to bacteria (Berlemont, 2017; Voshol et al., 2019). However, the variability of enzymes associated with CBMs or other accessory domains is much less in fungi than in bacteria (Talamantes et al., 2016; Berlemont, 2017), as exemplified here by the comparison of the genera *Aspergillus* and *Streptomyces*. In total 2540 different amylase and cellulase amino acid sequences with CBMs from *Aspergillus* and *Streptomyces* were retrieved from the databases, representing 385 different *Streptomyces* species and 60 different *Aspergillus* species. Amongst the sequences, approximately 90% of the GHs cellulase and amylase with CBM were identified from *Streptomyces.* In contrast, GH cellulases without CBM are more widespread in fungi representing about 80% of these cases. Thus, as a first difference between these two genera, many cellulases and amylases from *Aspergillus* lack any CBM, while *Streptomyces* enzymes contain many CBM types as well as a very diverse enzyme architecture associated with different CBMs (Figures 2, 3).

### Amylases With CBM From *Aspergillus* and *Streptomyces*

Among the starch degrading enzymes only two GH families are associated with CBMs in these two genera, the GH13 α-amylases and the GH15 glucoamylases. Neither *Aspergillus* nor *Streptomyces* contain genes encoding GH14 β-amylases with a CBM (Figure 2). As reported by the CAZy database and several studies, β-amylases are secreted mainly by plants, followed by several bacteria, and by a very limited number in fungi (Ray, 2004; Derde et al., 2012; Thalmann et al., 2019). Two CBM families were identified in bacterial β-amylases, i.e., CBM20 from species of *Bacillus* and *Clostridium* as well as CBM25 from *Paenibacillus*, and no CBMs were found in the fungal GH14 β-amylases (see review Janeček et al., 2019).

**FIGURE 4:**
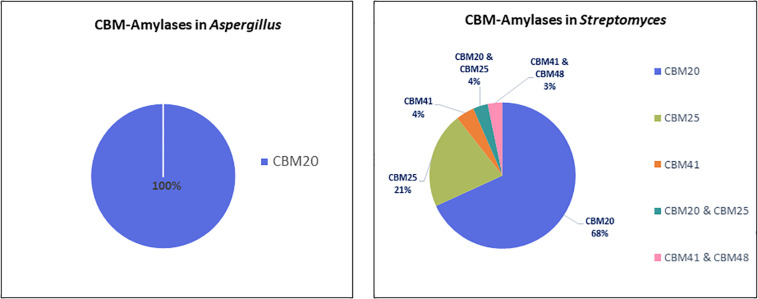
CBMs associated with amylases from *Aspergillus* (left) and *Streptomyces* (right).

In total, 606 protein sequences with starch binding CBMs were identified in *Aspergillus* and *Streptomyces* amylases. Among these sequences, more than half (466 sequences) are associated with CBM20. In *Aspergillus* amylases, CBM20 was found as the only starch binding domain associated with GH13 α-amylases and GH15 glucoamylases (Figure 2). More CBM families were found in conjunction with amylases from *Streptomyces*. CBM20 was found as the most frequently domain (68%), followed by CBM25 (21%), leaving the rest (around 4% each) to CBM41 and amylases with a combination of CBMs: CBM20&CBM25 and CBM41&CBM48. Thus, in amylases from these genera, CBM20 is the most dominant CBM (Figure 4).

In term of diversity of domain architecture, *Aspergillus* species tend to have a simple domain architecture consisting of only a single CBM20 module located at the C-terminus (Figure 2A). On the other hand, not only higher in the variability of CBM families, but also a more divers domain architecture of amylases with CBMs were identified in *Streptomyces.* For example, CBM20 and CBM41 are present as a single module, or multiple repeated domains from the same family. Moreover, some of the *Streptomyces* amylases have combinations of different CBMs attached: CBM20&CBM25 as well as CBM41&CBM48 (Figures 2B1,B2). In general, the binding modules of the CBM20 family are found in a single copy, while only in a few special cases a CBM20 is present together with other CBMs, such as CBM25, CBM34, and CBM48 (Janeček et al., 2019).

Based on the domain position, the CBM20s in α-amylases from *Aspergillus* and *Streptomyces* are always located at the C-terminal end (Figure 2). This is also true for other starch active enzymes, such as in GH14 (Oyama et al., 1999), GH15 (Bott et al., 2008; Roth et al., 2018), LPMO AA13 (Vu et al., 2014), and in most of the GH13 enzymes (see review Janeček et al., 2019). Based on the CAZy database, only in a few GH13s, the CBM20 is located at the N-terminal end of the catalytic domain, especially the GH13s from green algae (Lombard et al., 2014). Moreover, in the *Streptomyces*α-amylases, the CBM25 can be present either N- or C-terminally. Two copies of a CBM25 domain can be present at the N-termini, while co-occurrence of CBM25 with CBM20 at the C-terminus occurs. The other starch binding domains, CBM41 and the combination of CBM41 & CBM48, were found exclusively C-terminally.

Single or two copies of CBM41 can be found in several bifunctional enzymes, such as amylopullulanases (bifunctional with one catalytic center) and bifunctional α-amylase-pullulanases with two catalytic domains, one from α -amylase and one from pullulanase (Figure 2B). According to substrate binding studies, the CBM41 was demonstrated to be able to bind substrate with both 1,4- and 1,6 α-glucosidic linkages (Mikami et al., 2006; Van Bueren and Boraston, 2007). The CBM41s were largely identified in bacterial amylases and in only five members of eukaryotes, including four types of algae and a single species of fungi called *Ostreococcus tauri* (Lombard et al., 2014). CBM41s are often located at the N-terminus of the protein and present in two copies, while in a few cases, CBM41 is positioned C-terminally (see review Janeček et al., 2019). Interestingly, in bifunctional amylases from *Streptomyces*, CBM41 is present either as a single module or two repeated modules in between the amylase catalytic domain and the pullulanase domain (Figure 2B1), and even in combination with aCBM48 located C-terminal of the pullanase domain (Figure 2B2).

Other accessory domains present in the amylase architecture were annotated as Fn3, Glucodextran-N, DUF1966 and DUF 3372 (Figures 2A,B). In *Streptomyces*, the Fn3 domain is found in combination with CBM25. The CBM25-Fn3 domain combination is also present in *Microbacterium aurum* α-amylase and suggested to improve binding on starch indicated by the alteration of starch granule morphology (Huang et al., 2017). Furthermore, gradual domain truncations were made in the amylopullulanase from *Thermoanaerobacterium saccharolyticum* NTOU which consists of two Fn3 modules in between the N-terminal catalytic domain and C-terminal CBM20. The amylopullulanase remains active after removal of theCBM20 and the 2nd Fn3 module but further deletion of 1st Fn3 module that is connected with the catalytic module resulted in completely loss of activity (Lin et al., 2011). Generally, very little information is available on the function of the Fn3 modules in amylases. However, there is an indication that Fn3 is involved in binding between the enzyme and the polysaccharide substrates since its removal results in decreased or even loss of enzymatic activity. Another accessory domain, annotated as glucodextran_N in Pfam (El-Gebali et al., 2019) is especially found in bacterial and archaeal glucoamylases and glucodextranases (Mizuno et al., 2004). The domain consists of 17 antiparallel β-strands (Mizuno et al., 2004) which appear to stabilize its structure (Aleshin et al., 2003). This domain is absent in fungal glucoamylases. It was suggested that the eukaryotic glucoamylases may have evolved from prokaryotic glucoamylases together with substitution of the N-terminal glucodextran_N domain with the so-called peripheral subdomain and by the addition of a C-terminal starch-binding domain (Aleshin et al., 2003). The peripheral subdomain is an extra α-helix that is smaller than the other 12 α-helices in the catalytic domain structure and is located between α-helices αH10 and αH11 in the catalytic domain of glucoamylases *A. niger* (Aleshin et al., 1992, 2003; Lee and Paetzel, 2011). Lastly, several domains of unknown function, so-called DUFs, are present in the GH13 α-amylases of *Aspergillus* and the bifunctional α-amylase-pullulanase in *Streptomyces*, as DUF1966 and DUF3372, respectively. This domain folds in a β-barrel structure, however, the exact function has not been determined yet. Moreover, the truncation of this domain in the amylase from *Sclerotinia sclerotiorum*, ScAmy43, had no effect on the biochemical properties of the enzyme (Abdelmalek et al., 2009).

### Cellulases With CBM in *Aspergillus* and *Streptomyces*

Based on the datasets retrieved, various cellulase (GH) families do contain a CBM domain in *Aspergillus* and *Streptomyces* (Figure 3).

Interestingly, members of two GH families (GH7 and GH45) containing a CBM in *Aspergillus* are absent in *Streptomyces.* According to Segato et al. (2012), the genome of almost every *Aspergillus* encodes two GH7-cellobiohydrolases which can be present with or without a CBM. Meanwhile, three families, GH9, GH44, and GH48, containing a CBM present in *Streptomyces* are completely lacking in *Aspergillus*. The GH12 family proteins are present in both genera, but only in *Streptomyces* the GH12 have a CBM. The GH5 and GH6 cellulases with a CBM are found in both genera.

**FIGURE 5:**
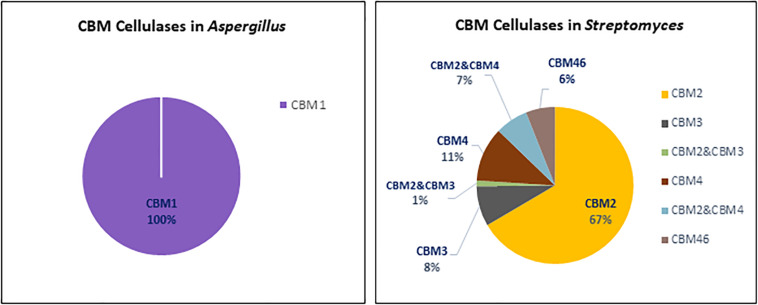
CBMs associated with cellulases from *Aspergillus* (left) and *Streptomyces* (right).

In the *Aspergillus* and *Streptomyces* genomes, 1946 sequences of cellulases with CBM domain were identified. Among these sequences 188 were annotated as cellulase with a single CBM in *Aspergillus*, 1256 as cellulase with a single CBM in *Streptomyces* and 502 as cellulases with multiple CBMs in *Streptomyces.* Figure 3 shows that *Streptomyces* cellulases are not only higher in their diversity of the CBM type, but also more varied in the domain organization compared to *Aspergillus.* In *Streptomyces*, four different CBMs associated with the GH cellulase catalytic domain were identified, including CBM2, CBM3, CBM4, and CBM46. Moreover, each CBM is not only present as a single module, but also appears in combination with other accessory domains including another CBM (CBM2&CBM4 and CBM2&CBM3). CBM2 is the most frequently found CBM (67%) among the four different CBMs in *Streptomyces* cellulases (Figure 5). In contrast to this diversity, in *Aspergillus*, CBM1 is the only module associated with GH cellulases and always present in a simple arrangement of one catalytic domain plus one CBM. Unlike observed for the amylases, the *Aspergillus* and *Streptomyces* cellulases do not share a common CBM.

The CBM1s are found almost exclusively in fungi, while the CBM2s are mainly found in cellulases from bacterial origin. Based on the domain structure and function, CBM1s are relatively small, consisting of approximately 36 amino acids with 4 conserved cysteine residues involved in the formation of two disulphide bridges required for protein folding (Mattinen et al., 1997). The fold of CBM1 is called a cysteine knot (Boraston et al., 2004). This knotted arrangement is usually associated with a β-sheet structure and highly conducive to protein stability (Cheek et al., 2006). In contrast, the bacterial cellulose binding modules like CBM2 are usually much larger in size, about 110 amino acid residues with a β-sandwich fold providing the ligand recognition site (Boraston et al., 2004). In general, a regular plane surface with three aromatic residues such as tyrosine, as well as polar residues glutamine and asparagine are involved in CBM1-cellulose binding (Beckham et al., 2010). Likewise, the CBM2 β-sandwich fold presents a planar surface containing conserved aromatic residues, usually tryptophan and tyrosine, involved in substrate binding. Although the CBMsin *Aspergillus* and *Streptomyces* are different in protein structure, creating a chimeric enzyme by fusing a bacterial CBM with a fungal cellulase has potential to create an active enzyme. As reported by Voutilainen et al. (2014), fusing a bacterial CBM3 to a GH7 cellobiohydrolase from the thermophilic fungus *Talaromyces emersonii* which in fungi is often associated with a CBM1 domain, resulted in an active chimeric GH7 with higher thermal stability.

CBM1s are mainly located at the C-termini of GH5, GH7, and GH45. Only in the GH6 of *Aspergillus*, the CBM1 is positioned at the N-terminus (Figure 3A). Similar domain structures are also present in other fungi such as *Trichoderma* (Rahikainen et al., 2019). In a few special cases multiple CBM1 domains are present in a single protein. For example, five different CBM1 modules are arranged in tandem at the N-terminus of GH45 endoglucanase from the yeast, *Pichia pastoris* GS115 (Couturier et al., 2011). Furthermore, in *Streptomyces* the CBM2 is present either at the N- or C-terminus of GH cellulases. Among those, 76% were detected at the N-terminus of the cellulase catalytic domain, such as in CBM2_GH5, CBM2_GH6, and CBM2_GH48. The remaining 16 and 8% of the CBM2 were found at the C-terminus, connecting one catalytic domain with other accessory domains or different CBMs (Figures 3B1,B2, respectively). A CBM2 is widely occurring in cellulases from bacteria but much rarer in eukaryotes with only a few examples from gastropods and nematodes.

In the cellulase domain organization, CBM3 is always located at the C-terminus of the catalytic domain and represents the second most frequently found CBM in the domain architecture of cellulases (Figure 3B). Besides its presence as a single module, CBM3 is also commonly found in a multidomain architecture. In *Streptomyces*, CBM3s were mostly associated with GH6 in co-occurrence with Fn3 domains and linked with CBM2 in the GH9 multidomain architecture. A CBM3-Fn3-Fn3-CBM2 architecture was also found in the *Thermobifida cellulosilytica* TB100 GH9-endoglucanase (Tóth et al., 2017). A CBM3 domain consists of 150 amino acids and has a β-sandwich fold with nine strands and one calcium binding site (Tormo et al., 1996; Shimon et al., 2000).

In contrast to CBM3, a single CBM4 domain is always present at the N-terminus in the GH6 and GH9 family from *Streptomyces* (Figure 3B1). In the GH9 multidomain architecture, CBM4 can also be present together with CBM2 and another accessory domain, CelD-N. In general, the CBM4 modules are found in xylanases and endoglucanases in agreement with their binding affinity toward either xylan or β-glucan (Abou-Hachem et al., 2000; Boraston et al., 2002; Simpson et al., 2002). Several binding studies with CBM4 showed differences in substrate binding preference. The differences in substrate binding affinity of CBM4 is determined by the length and topography of the surface loops. The long shallow groove conformation in CBM4 *Cellulomonas fimi* provides better binding to 1,4-β-glucan than 1,3-β-glucan (Coutinho et al., 1992).,The prominent U-shape with high-sided walls formed by loops between the β-strands from *Thermotoga maritima* CBM4 contribute to the higher affinity to 1,3-β-glucan (Zverlov et al., 2001). The conserved aromatic clusters in CBM4 containg tyrosine and/or tryptophan contribute to the surface binding site and are suggested to be involved in binding specificity (Boraston et al., 2002). Furthermore, replacing the CBM1 of a fungal acetyl xylan esterase with the CBM4 from the thermophilic bacterium *Rhodothermus marinus* Xyn10A enhanced the specific activity and the thermostability of the acetyl xylan esterase (Liu and Ding, 2016). Indicating that creating a chimeric enzyme between a mesophilic carbohydrate esterase and a CBM4 from a thermophile does result in improved thermophilic properties.

Another CBM present in *Streptomyces* cellulases is CBM46. According to the CAZy database, CBM46 is exclusively found in bacterial cellulases. In GH cellulase family 5, the CBM46 is present in combination with an accessory module called CBM_X2 (PF03442). A similar architecture was also found in GH5s of *Bacillus halodurans* which are associated with CBM46 and CBM_X2 (Venditto et al., 2015). The CBM_X2 or generally also called the X2 module is the immunoglobulin (Ig)-like domain with capability to bind cellulose and bacterial cell walls (Mosbah et al., 2000; Kosugi et al., 2004; Liberato et al., 2016). Moreover, the catalytic activity of GH5 from *Bacillus licheniformis* is entirely dependent on its CBM46 and X2 modules (Liberato et al., 2016). Besides in GH5, the X2 module is also present in GH74 endoglucanase from *Paenibacillus polymyxa* A18 with the arrangement GH74_X2_CBM3. Based on the enzyme activity studies, endoglucanase with X2-CBM3 domains have a several fold increase in activity toward insoluble substrates. In substrate binding studies, the module X2 showed a higher affinity toward phosphoric acid swollen cellulose, whereas CBM3 showed a higher affinity toward crystalline cellulose (Avicel) (Pasari et al., 2017).

### Other Accessory Domains: The Fn3 Domain

Other types of accessory modules found in *Streptomyces* GH cellulases are the Fn3 domain and the variants of the X2 module CelD-N and Big7. According to the Pfam database (El-Gebali et al., 2019), domains annotated as X2, CelD_N and Big7 are a group of bacterial Ig-like domain which are often associated with bacterial GHs cellulases and suggested to be involved in assembling multienzyme complexes called cellulosomes. The Fn3 domain is a domain composed of a seven-stranded β-sandwich, usually occurring in multiple copies in both intracellular and extracellular proteins.

Further investigation of the presence and role of a Fn3(-like) domain in GH cellulases, was performed by querying the genomes of both *A. niger* and *S. leeuwenhoekii*, as representatives of the two genera we have focused or research on and mapping the Fn3 domains present in these and other proteins involved in polysaccharide degradation (Table 1). Indeed, besides cellulases there are several proteins with Fn3 domains, including GH3 β-glucosidase, GH3 β-xylosidase, GH5 mannosidase, GH18 chitinase, and chitin biosynthesis proteins with several types of domain organization. This wide occurrence of Fn3 domains, suggests that the Fn3 domain could be of functional significance.

**TABLE 1:** Protein domain associated with Fn3 in *A. niger* and *S. leeuwenhoekii.*

Organism	Domain associated with Fn3	Main activity	Accession number (NCBI)
*A. niger*	GH3_Fn3-like	β-glucosidase	CAK48740.1
	GH3_Fn3-like	β-xylosidase	CAK37179.1
	RhgB__Fn3_CBM-like	Rhamnogalacturonat lyase	XP_001400741.2
	Chs5n_Fn3_BRCT-CHS5like	Chitin biosynthesis protein	XP_001395839.2
*S. leeuwenhoekii*	GH5_Fn3_CBM2	Mannosidase	CQR60862.1
	CBM2_Fn3_GH18	Chitinase	CQR61740.1
	CBM4_Fn3_GH18	Chitinase	WP_029384933.1

As can be seen from Table 1, Fn3 is often present between the catalytic domain and a CBM in *Streptomyces*, in correspondence to the suggestion that it plays a role as stable linker between the enzymatic and substrate binding domain (Jee et al., 2002; Valk et al., 2015). However, the Fn3 domain in *Aspergillus* GH3 is positioned at the C-terminal end, and not followed by a CBM. Several other fungal β-glucosidases carry Fn3 domains, such as those from *Aspergillus aculeatus* and *Trichoderma reesei* (Suzuki et al., 2013), as well as the thermophilic fungus *Rasamsonia emersonii* (Gudmundsson et al., 2016).

Although Fn3 is suggested as a stable linker (Jee et al., 2002), many studies showed that the Fn3 domain also has relevance to a ligand binding function and is involved in protein-protein interaction (Hansen, 1992; Koide et al., 1998). From scanning electron microscope studies, it is concluded that Fn3 present in a bacterial cellobiohydrolase contributes to cellulose surface disruption, and therefore the presence of Fn3 increases the efficiency of degradation (Kataeva et al., 2002). Similarly, it has also been demonstrated that an isolated CBM2 from *Cellulomonas fimi* cellulase disrupted the surfaces of cellulose fibers (Din et al., 1991), indicating a potential functional similarity between Fn3 and a genuine CBM. Moreover, removal of the Fn3 domain can result in decreased or loss of enzymatic activity, as we found in a study on the GH3 function (Figure 8). As other examples, removal of the Fn3 domain of a xylanse dramatically decreased xylanolytic activity from *Cellulosimicrobium* sp. strain HY-13 (Kim et al., 2009), *Flavobacterium johnsoniae* (Chen et al., 2013), a *Marinifilaceae* bacterium strain SPP2 (Han et al., 2019) and cellulase activity for the GH6 cellobiohydrolase in another bacterial species (Cerda-Mejía et al., 2017). Deletion of the Fn3 domains also resulted in reduced enzymatic activity of GH55 endoglucanase (Conway et al., 2016) and GH9 endoglucanase (Zhou et al., 2004). Likewise, a CBM-truncated enzyme, in some cases, showed a significant loss of enzymatic activity, such as in GH9 (Burstein et al., 2009) and GH5 (Zheng and Ding, 2012). Surprisingly, our own results suggested that designing a GH3 chimeric enzyme by replacing the Fn3 domain with a *Streptomyces* CBM2 domain does not result in a functional GH3 enzyme (Figure 8, Sidar et al., in preparation). Thus, a function of the Fn3-like domain may be both to potentiate the activity of the GHs and to increase the substrate proximity to the catalytic domain in a similar fashion as the action of a CBM. Similar as found for CBM1 and CBM2 (see above) the (C-terminus of the) Fn3 domain consists of clusters of aromatic residues, indicating that it could indeed play a role in ligand binding interaction, instead of solely existing as a linker (Lima et al., 2013). Another similarity between Fn3 and CBM is that some CBMs have a binding site for calcium which plays a significant role in the substrate interaction (Tormo et al., 1996; Montanier et al., 2010; Yaniv et al., 2012). Kataeva et al. (2002) also experimentally showed that each Fn3 domain in cellobiohydrolase CbhA binds calcium. The literature evidence for the Fn3 domain performance so far suggest a putative dual role of this domain. We hypothesize that Fn3/Fn3-like domains play a biological role both in potentiation the functionality of the catalytic center and as a functional domain, rather than merely as a simple spacer, and may even represent a novel CBM-like domain.

## Designing New Chimeric Enzymes Inspired by the Domain Architecture

In the field of enzyme engineering, the design of chimeric enzymes has been developed as one of the promising approaches to obtain an enzyme with the desired characteristics like improved hydrolysis efficiency. A chimeric enzyme is commonly generated by fusing the GH catalytic domain obtained from one species with another protein domain such as a CBM from another species. In several studies, chimeric enzymes have been shown to improve the catalytic efficiency, thermostability, as well as substrate specificity (Oliveira et al., 2015; Saadat, 2017; Christensen et al., 2020; Gilmore et al., 2020).

As shown above *Streptomyces species* display a much higher diversity in cellulase domain architecture than *Aspergillus*. This observation led us to review the *Streptomyces* domain architecture in more detail as the basis for design of novel chimeric enzymes with cellulolytic activity. As an example, we selected *Streptomyces leeuwenhoekii* C34 (Busarakam et al., 2014) and *Aspergillus niger*.

**FIGURE 6:**
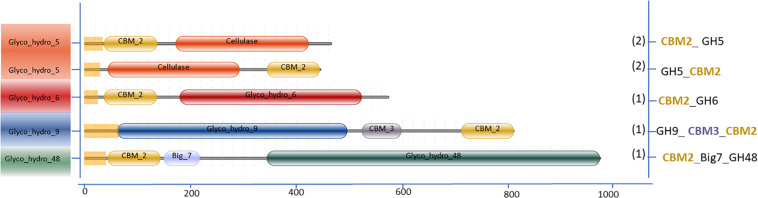
Cellulases associated with CBM2 in *S. leeuwenhoekii* C34.

**FIGURE 7:**
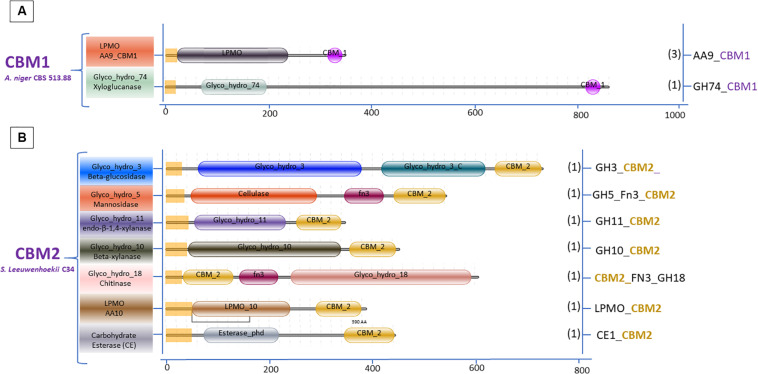
Domain organization of polysaccharide degrading enzymes other than cellulases associated with two dominant CBMs: **(A)** CBM1 *A. niger* 513.88 and **(B)** CBM2 *S. leeuwenhoekii* C34.

The variation in the domain organization of cellulases tethered to a CBM in *S. leeuwenhoekii* is presented in Figure 6. The cellulases of the GH5, GH6, GH9, and GH48 families are all associated with CBM2. When expanding our genome mining efforts in *S. leeuwenhoekii*, to proteins associated with a CBM2, other than cellulases, a considerable group of enzymes such as xylanases, chitinases, LPMOs and an esterase showed up from the genome sequence, in addition to a GH3 family β-glucosidase (Figure 7). The same mapping of enzymes containing a CBM1 in *A. niger*, resulted in only two other proteins, both related to polysaccharide degradation, being a cellulose active LMPO and a GH74 xyloglucanase (Figure 7).

**FIGURE 8:**
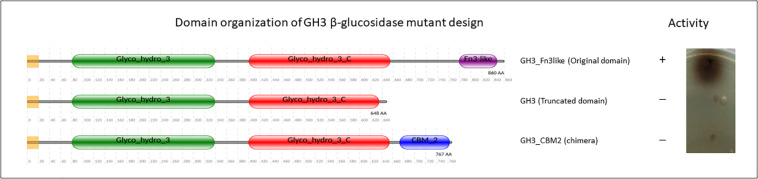
GH3 β-glucosidase activity determined using an agar plate assay with esculin as substrate (Pointing, 1999).

In fungal cellulolytic enzyme cocktails, the β-glucosidases are considered to be the limiting factor in obtaining full conversion of cellulose to glucose. The GH3 family β-glucosidase in filamentous fungi do not have native CBM domains (see Figure 3). Therefore, we suggest that based on the *Streptomyces* domain architecture, a new concept to design chimeric enzymes could be studied by fusing a CBM2 domain from a *Streptomyces* GH3 β-glucosidase to a well-known *A. niger* GH3 β-glucosidase. However, it should be kept in mind that the design of new chimeric enzymes is not as simple as replacing one domain for another as shown by the example of replacing a Fn3 domain by CBM2 (Figure 8 and section “Other Accessory Domains: The Fn3 Domain”).

## Future Perspective

As functional domains, the CBMs have attractive characteristics like promoting substrate binding and thus supporting the catalytic function of enzyme. So far, CBMs have been classified in more than 80 families and more CBM families will undoubtedly be discovered. CBMs appear in association with a wide range of proteins in particular the CAZymes and are assembled in various domain organizations in numerous microorganisms. As an example, the diversity in the domain organizations in *Aspergillus* and *Streptomyces* is presented in this review. The *Streptomyces* species harbor cellulases and amylases with a more diverse domain organization, including CBMs and other accessory domains, than found in *Aspergillus*. This remarkable feature provides inspiration to design chimeric enzymes with a potential for substrate degradation improvement.

Fusions of enzyme domains have been developed in many scientific studies to create improved chimeric enzymes. In one of our studies, the *Aspergillus* GH3 enzyme that does not have a CBM but does have a C-terminal Fn3-like domain, was redesigned to replace the Fn3 with a CBM2 of *Streptomyces*. To our surprise these studies revealed that the Fn3-like domain of the fungal GH3 seems to play a pivotal role in the enzymatic activity (Figure 8), This observation might indicate that in addition to the well-known CBMs more domains could play an important role in the activity of CAZymes. Clearly, creating chimeric enzymes by modifying domain arrangement could provide advantages for enhancing enzymatic activity. Overall, the variability in the CBM domain organization of cellulases and amylases, as shown in this review, offers new opportunities to rethink the strategy for designing multiple-domain enzymes. This approach is not limited to chimeric cellulases and amylase to improve degradation of recalcitrant substrate but also holds promise for other CAZymes such as xylanases, pectinases, and oxidative enzymes.

## Author Contributions

AS analyzed the data, wrote the review, performed the literature data mining, and designed the figures. EA conducted the cellulases analysis of *Streptomyces*. GV performed the bioinformatic analysis for the graphical domain annotation. AR presented ideas and edited the review. EV and PP involved in the review outline, ideas, planning, supervision, writing, and editing. All authors reviewed and agreed on the final manuscript.

## Conflict of Interest

EA was employed by company Sun Pharmaceutical Industries Europe BV. GV and PP were employed by company Dutch DNA Biotech B.V. The remaining authors declare that the research was conducted in the absence of any commercial or financial relationships that could be construed as a potential conflict of interest.
